# Case of Ovarian Tumor, Successfully Removed by Extensive Abdominal Section, and Recovery of the Patient

**Published:** 1856-12

**Authors:** Nelson Winton

**Affiliations:** Havana, Schuyler County, N. Y.


ART. III.— *Case of Ovarian Tumor, successfully removed by extensive Abdominal Section, and recovery of the patient.* By NELSON WINTON, M. D., of Havana, Schuyler County, N. Y.

As the case, in reference to an operation, presented many discouraging features, a brief previous history of it is deemed advisable. As no notes were taken preceding the removal of the tumor, the early history of the case is gathered from my recollection, prompted by that of the patient.

Miss Hannah Wing, is a maiden lady, 38 years of age, industrious in her habits, cheerful in her disposition, intelligent, and of strong mind.

In July, 1842, she was attacked with bilious pneumonia, followed by vomiting of green acid bile, which excoriated her lips and skin wherever it came in contact with them, and produced effervescence by throwing sup. carb. of soda into the ejected matter.

I saw her about forty-eight hours after the attack, when she had just taken Lee's purgative pills, which much increased the vomiting; I found her with all the symptoms of acute gastritis, and scarcely able to keep anything on the stomach. Venesection, sinapisms, and epispastics, were resorted to. Various internal remedies were instantly ejected, when all attempts to give medicine by the mouth were abandoned for several days.

She was directed to take iced water freely, sub. morph. by the mouth, and in the forms of enema when it could not be retained on the stomach. At the end of about eight days, she retained a small quantity of wine diluted, and also some toast water. After taking blue mass and sul. quinine, she was convalescent.

At intervals, varying from one to two years, she has had attacks of bilious vomiting and diarrhœa, attendant on dyspepsia, which usually subsided after a cathartic of calomel, followed by an opiate.

In Dec., 1850, she had typhoid fever for four weeks, which at that time was prevalent in this village.

In May, 1852, she had another severe attack of green bilious vomiting, but the stomach was not as irritable, nor the excretions as mixed, as in 1842. The pain was relieved by venesection, opiates, the warm bath generally, and the hip bath; after which, a cathartic dose of calomel, followed by castor-oil, which was rejected, and the stomach becoming irritable, all attempts to give medicine by the mouth were abandoned for several days; enemas of various kinds were prescribed without effect, and for eighteen days an obstinate constipation ensued. For eight days nothing was taken or retained on the stomach, except iced water, and this but for a short time; it however allayed thirst and pain in some measure, besides rinsing off the stomach The bowels were greatly distended, particularly the colon on the left side, though the surface was not as tender as might have been expected from the obstinate constipation, depending, as I considered it, on the inflammation of the colon.

As soon as the stomach would bear it, various cathartics were tried, and finally discharge was produced, by throwing up the rectum a large quantity of warm water with an injection pump; speedy relief followed this operation.

Soon after this a tumor, about the size of a teacup, appeared in the left hypogastric region, which I pronounced an ovarian tumor.

The abdomen began to distend, and soon it was evident that ascites existed; the usual remedies, such as cathartics, diuretics, etc., were prescribed, without any benefit.

Almost every diuretic, in and out of the materia medica, was tried, without increasing the urinary discharges, which secretion was scanty, from the appearance of the tumor, up to the time of its removal.

Menstruation was generally regular; she was able to follow her avocation as nurse, till June, 1854; the abdomen was much distended; there was great difficulty in breathing; the stomach was very irritable at times, and a diarrhœa existed, the consequence of indigestion; she also had colic and pleuritic pains.

July 23, 1854, she measured forty-one inches around the abdomen, when paracentesis abdominis was first performed, and sixteen pounds albuminous fluid were drawn off, being of the consistence and appearance of the white of an egg. This operation reduced her measure to thirty-one inches. Paracentesis produced great relief for some weeks, until the same symptoms recurring, it was again resorted to with like result.

The following are the dates of, and quantities removed by paracentesis:

| | | |
|---|---|---|
| 1854. | July 23d, - - - - - - | 16 lbs. |
| | Sept. 30th, - - - - - - | 12 " |
| | Dec. 18th, - - - - - - | 9½" |
| 1855. | March 3d, - - - - - - - | 13 " |
| | May 24th, - - - - - - | 11 " |
| | July 8th, - - - - - - - | 13 " |
| | August 16th, - - - - - - | 7½" |
| | Oct. 30th, - - - - - - - | 8½" |
| | Nov. 30th - - - - - - | 12 " |
| 1856. | Jan. 11th, - - - - - - - | 10 " |

| | |
|---|---|
| March 3d, - - - - - - - | 12 lbs. |
| April 28th, - - - - - - - - | 9¼" |
| May 25th, - - - - - - - | 12 " |
| June 22d, - - - - - - - - | 11 " |
| July 12th, - - - - - - - | 9½" |
| July 21st, - - - - - - - - | 9 " |
| August 7th, - - - - - - - | 11 " |
| August 21st, - - - - - - - | 11 " |
| Sept. 1st, say - - - - - - - | 13 " |

About one year after the tumor was discovered, apparently another made its appearance on the opposite side, followed by three others, one above, another below, the tumor on the right side; and a third below the first on the left side; all these were immovable, but subsequently were found to be parts of the same tumor.

1856. January. The first tumor had much increased in size; had become tender to the touch and extremely painful, attended with vomiting of vitiated bile, as before; twenty grains of calomel, with half an ounce of castor-oil, removed free bilious fæces, but not allaying pain, opiate enemas were administered with much relief for a time.

The stomach rejected everything taken, so that only iced water was used.

Again an obstinate constipation occurred, which continued three weeks. Cathartic enemas causing severe and excruciating pain in the rectum, only opiate enemas were used, and even these gave considerable pain each time for a short period.

About nineteen days after pain in the tumor commenced, it disappeared, and a cavity was apparent; two days after this the bowels moved spontaneously, and vomiting ceased. Toward the latter part of this attack, vomiting had been easy, more like water brash.

Her strength was much reduced, but after about a week it was in a measure regained.

After paracentesis had been performed two or three times, she inquired if any remedy could effect a cure. I informed her that nothing would short of *ovariotomy*: at the same time explaining its nature and danger, and the success and failures attending such operations, as reported in the medical journals of the day. I proposed the operation to her, to which she replied, that she would never submit. I did not urge her then nor since.

In June last, her extreme suffering induced her to urge me to remove the tumor. She said she had for some time reflected on the subject, and had concluded to have it removed. I informed her that the chances of success were greatly lessened since I proposed it in 1854, on account of her impaired health, and the increased size of the tumor; besides, the weather was unfavorable, and that I feared, from so much inflammation, firm adhesions had formed, between the tumor and adjacent parts; but she continued to importune me on the subject, and I promised her that when the time was suitable I would undertake it, designing to wait till the cool weather of October, but her health beginning to fail, with rapid emaciation, I feared that she would not survive until that time. The heart was pressed up opposite the third and fourth ribs; respiration was extremely difficult; and the abdomen was enormously distended. I told her that I would request the medical gentlemen in this and the neighboring villages, to see the case, and would be guided by their decision as to the propriety of an operation.

On the 21st of August, nine or ten of the medical faculty met, when it was requested by several of them, that the ascitic fluid be removed, in order to afford a better opportunity of examining the tumor or tumors. To this I reluctantly consented, preferring to leave the fluid in the cavity of the abdomen, in order to prevent irritation of the viscera while examining and seperating adhesions, should they exist. On removing the fluid an operation was decided on, many consenting to it, solely on account of the patient's great anxiety on the subject.

I then proposed delaying the operation until the ascitic fluid had formed again, for these reasons: first, that the weather would be cooler; and second, that the patient was much wearied and exhausted by the operation of tapping.

Six hours after paracentesis, she had violent pains in the abdomen and chest, when an opiate-enema was given, and her sufferings being very severe, in fifteen minutes afterward it was repeated by her anxious friends; in forty-five minutes she became easy, but by this repetition of the anodyne, she had taken an over dose of morphine, causing vomiting, which continued till the next evening, when it ceased, and she remained in a comfortable state for the next seven days; pain and difficult breathing then supervened, and continued until the removal of the tumor.

Sept. 1st, Drs. Wey, Hinman, Brown, Fish, Pratt, Agard, Wager, Mead, and E. D. and L. S. Bailey being present.

The patient was placed on a narrow table for the operation. She had increased in circumference, nine inches, in eleven days. Marks were made across the linea alba, with nit. silv., as a guide for the sutures. Chloroform was administered by Mr. Covel, medical student, in a very careful manner.

At 10 o'clock, A. M., standing on the right side of the patient, I cut down to the peritoneum, commencing about two inches below the ensiform cartilage, and carrying the incision to near the pubes. Slight hæmorrhage was arrested by sponge and cold water.

I divided the peritoneum with a scalpel, sufficiently to introduce first two fingers of the left hand, as a guide, and to protect the contents of the abdomen. Slight adhesions along the linea alba were separated by the fingers; a good deal of ascitic fluid escaped when the peritoneum was cut through.

This section disclosed an enormous multilocular tumor, into one of the cysts an exploring needle passed, and albuminous fluid escaped. The tumor extended from the diaphragm (which it pressed up two inches) down into the pelvis, completely covering the viscera. The omentum adhered on the left side of the tumor, over a surface six inches square. This I requested Dr. Wey to separate, which he did with the finger; at the same time I separated an adhesion on the right side of the tumor, some three inches square, after this the tumor was slightly raised by Drs. Wey and Fish, when I passed my hand under the base, and found an adhesion about the size of my hand, which I easily broke up with the finger, except about two square inches: this was so firmly attached that, at first, I apprehended there were two pedicals; adhesion was separated as before.

When Drs. Wey and Fish raised the tumor, I passed a ligature, composed of four stout silk threads, around the pedicle, which sprang from the broad ligament of the uterus on the right side, with the thumbs and index fingers I pressed it down to the broad ligament, and requested Dr. Agard to tighten it with great force, in order to prevent danger from hæmorrhage; I took hold, also, and used great additional strength.

I then passed a tenaculum through the pedicle, above the ligature, severed it with a probe-pointed bistoury, and the tumor was lifted out of its bed by Drs. Wey and Fish; on examining the severed pedicle, not the least hæmorrhage was perceptible. I also satisfied myself that no tumors remained; not over four ounces of blood was lost in the operation.

Dr. Wey removed the ascites fluid, remaining in the abdominal cavity, with an elastic tube attached to a common syringe, and washed off the blood from the omentum. I then replaced it in its natural position, requesting Dr. Wey to close the incision with interrupted sutures, including the peritoneum; this was done on account of apprehended vomiting, and the sequel fully justified this precaution. Between the sutures were placed adhesive straps, eighteen inches long, and others diagonally over them. At this stage of the dressing, severe vomiting, as was feared, ensued. I immediately placed my hands on each side of the abdomen, and it required all the strength I could apply to keep the dressing in place, on account of the unusually severe contractions of the abdominal muscles. Food, which she had taken five hours previous, was thrown up without the least appearance of digestion.

I had previously prepared a bandage of stout cotton cloth, of sufficient width to extend from the sternum to the pubes, with gussets on each side, in order to fit it to the hips, and a piece of whalebone on each side of the abdomen, to keep it in place; on each side were six straps, with buckles on one side.

As soon as vomiting abated sufficiently, compresses of cotton wadding were placed on each side, and over these one of muslin; the bandage was placed over the whole and buckled as firmly as the patient could bear. She was placed in bed a few minutes before 11 o'clock, A. M. The tumor was composed of small cysts or hydatids, varying from half of an inch to three inches in diameter, containing albuminous fluid, and weighed twenty pounds, making, with the ascitic fluid, thirty-three pounds.

It should be mentioned that Miss Wing had had an enema of $\frac{1}{3}$ grain sul. morph. administered every eight hours, for eight or nine weeks. For the last three weeks it has been discontinued, and she has been equally comfortable without it. The time occupied in removing the tumor was under thirty minutes, though I think with the venerable DeLamater, that an operation that is well done is soon enough done.

She has had frequent attacks of vomiting and diarrhœa, caused invariably by indigestible food, causing great emaciation and considerable debility.

Sept. 1st, 11, o'clock, A. M. Vomiting severe, throwing up undigested food; severe pain in abdomen; pulse 90, and small; extremities cold; want of air, requiring constant use of a broad fan, although a free current of air was passing over her, the doors and windows being open.

Ordered an enema of half a grain sul. morph., dissolved in one ounce of water, every hour, until relief of the pain.

2 o'clock, P. M. Extremities warm; pulse 120, rather fuller; much pain in the abdomen and chest; severe vomiting of greenish fluid.

Gave her freely of iced water to rinse off the stomach and allay thirst.

4 o'clock. Much relieved from pain; discontinued enema; pulse 110; vomiting continues.

Sept. 2d, 6, A. M. Pulse 95; tongue rather dry, not coated; has had a sleepless night; stomach rejecting all fluids taken into it; at times severe pain in chest and abdomen; heart remaining in its natural position.

Ordered an enema of sulph. morph.

12, noon. Pulse 105; vomiting continues; pain much relieved.

6, P. M. Pulse 110; vomiting continues.

Sept. 3d, 6, A. M. Pulse 95; passed a tolerable night; slept some. At 11 o'clock, vomited about one pint of green watery fluid, which gave much relief, has not vomited since; great restlessness, asked to be turned on right side, could not lie on it but a few moments, nor on left side a moment. Asked for food, took codfish soup and toast.

11, A. M. Severe pain in left side under false ribs, was relieved by enemas of sulph. morph.; bowels somewhat distended with flatus; want of air requiring constant use of fan; one pint of water was thrown up the rectum, producing a small discharge, which the nurse said resembled meconium. This gave some relief. Asked to be turned on side—could lie on right but not on left side; heart resuming its natural position. I told her the heart was getting home again: she said she thought it had got there, and had appeared so for some hours.

4th day. Pulse 105; had some sleep; countenance much improved; said that she had not for a long time had as much confidence of getting well as now; asked for food; a minute quantity of mutton chop, bread and butter, codfish soup, and black tea, was taken by her. Turned on her side by her own effort, and could lie for a few minutes on either side.

5th, 6, A. M. Pulse 110; warm day, thermometer 90; had rested tolerably well; could turn herself in bed without difficulty, or much pain. Ate mutton chop, tomato, and peaches, too freely; pain in right side under false ribs. Morphine enemas gave much relief.

Noon. Pulse 125; skin hot and dry; vomiting had returned, ejected peaches not masticated; countenance bad; lifted her on sheet to W splint; applied cold water to body and upper extremities and a pediluvium to the feet.

4, P. M. Pulse 120; copious sweating came on, which gave me great anxiety; fanned her with sheet and rubbed the body with a wet cloth.

6, P. M. Pulse 116, soft and full; skin dry; severe pain in abdomen, opiate enema gave relief; continues to throw off the stomach drinks tinged with green.

6th day, 6, A. M. Pulse 88; slept a good deal; turns from side to side without difficulty; called for food; countenance good; is much encouraged, thinks she will get well; gave orders about her domestic affairs.

10, A. M. Pulse 100; constant desire to evacuate the bowels, attended with tenesmus.

An enema of $\frac{1}{6}$ grain of sulph. morph., instead of $\frac{1}{3}$, as prescribed, was administered, and repeated after one hour, without relief.

Noon. Pulse 98. $\frac{1}{3}$ grain sul. morph. was injected into the rectum, and gave great relief.

Directed that $\frac{1}{3}$ grain sulph. morph, dissolved in one ounce of water, be used as an enema every eight hours, until otherwise ordered. A bloody serous discharge, very offensive in smell, from the wound where ligature of pedicle was brought out.

7th day, 6, A. M. Pulse 90; has passed a comfortable night, after vomiting a dark thick greenish fluid; vomiting continued till noon, when she took rye mush, this being the first food she had retained for over twenty-four hours. Warm day, thermometer 90.

On account of the edges of the wound being swollen, and unusually red, I removed the next above the lowest suture, having some fears of erysipelatous inflammation.

9, P. M. Pulse 98; has slept pretty well; no vomiting since noon. She was put on the splint, and placed on the bed at 3, P. M.

8th, 6, A. M. Pulse 80; has passed a comfortable night; some pain in the back; discharge from incision not as offensive in smell; vomited but once, a green watery fluid; retains rye mush; tongue red and dry as at former periods, a slight aphtha is making its appearance; a small tumor in upper part of incision; removed the two upper sutures, placed on adhesive straps.

12 o'clock, noon. Pulse 90; comfortable.

9th, 6, A. M. Pulse 75; tongue red and dry, with aphthous spots more distinct.

4, P. M. Pulse 85; removed straps and part of the sutures; skin natural and symptoms favorable.

10th. Patient doing well; pulse 80; no unfavorable heat or feverishness; some discharge from wound.

11th. Pulse 89; removed all remaining sutures, and union by first intention, except half an inch above umbilicus, where the sutures were farthest apart, and about half an inch in lower part around ligature.

12th. No change.

Being obliged to be absent from town, I left her in charge of Drs. Goodrich, of Brooklyn, and Hinman, of this village, for two days.

13th. Doing well; pulse 80; urgent desire to evacuate the bowels; warm water thrown up the rectum produced free evacuations of fœcal matter; enema of sulph. morph. have been regularly given every eight hours since sixth day.

14th, 8, A. M. Pulse 76; has slept well during the night; aphthæ less; says she has not rested as well, any night, for the last six months; takes food of broiled squirrel and oyster soup.

15th. Pulse 88; pain in the abdomen; had four or five motions of bowels, somewhat copious, the first two offensive in smell.

16th. Pulse 90; has had some sleep.

17th. Pulse 85; rested well; aphthæ better.

18th. Pulse 88; rested tolerably; some colic pains.

I was obliged to go to Owego to see a sick sister, and left her in charge of Dr. Hinman.

19th. Dr. H. dressed the wound, and placed the ligature under adhesive straps; afterward feeling some irritation in the wound, she directed her nurse to raise the lower strap, when the ligature came away.

20th. Pulse 80; has had an attack of cholera morbus, vomiting yellow bile of the consistence of the yolk of an egg; vomited four times, and had four motions of the bowels.

21st. Pulse 78; has rested well; no vomiting or diarrhœa; takes food and retains it. She walked into front room at two different times, and sat an hour in a rocking chair.

22d. Pulse 78; she had a good night's rest; ate a hearty breakfast; at noon had severe pain in the abdomen, much flatus, and a large dejection from the bowels.

23d, 8, A. A. Has rested tolerably well; appetite good; aphthæ better, colic pains and flatulency; wound nearly healed.

24th. Pulse 72; comfortable, except colic pains occasionally; appetite tolerable; wound healed.

25th. Pulse 72; appetite tolerable; some colic pains; opiate enemas given every seven hours.

26th. Rested tolerably.

27th. Pulse 76; comfortable; walked into front room and back several times; appetite tolerable.

28th. Same.

29th. Pulse 80; rested well; very comfortable.

Oct. 7th. Has had occasional vomiting and diarrhœa during the past week. Dyspepsia, her old enemy, has been troublesome, but to-day she is very comfortable.

Nov. 19. It is now over eleven weeks since the removal of this tumor, and so far as this operation is concerned, I consider it perfectly successful.

For the last fortnight she has had but one attack of this kind, and it was caused by round clams and wild duck.

She is now rapidly gaining in strength and flesh. She walks around and rides out daily from two to seven miles. Her appetite is good, and has l een for some time.

